# Practicing Active Control of Thumb Force Alters the Excitability of Anterior Horn Cells of the Spinal Cord

**DOI:** 10.7759/cureus.87695

**Published:** 2025-07-10

**Authors:** Ayato Mizoguchi, Katsunori Kiyohara, Naoki Kado, Toshiaki Suzuki

**Affiliations:** 1 Graduate School of Health Sciences, Kansai University of Health Sciences, Kumatori, JPN; 2 Clinical Research Division of Rehabilitation, Sakakibarahakuho Hospital, Tsu, JPN; 3 Center for Research and Education, Kobe College of Rehabilitation and Health, Kobe, JPN

**Keywords:** force control, f wave, motor adaptation, motor neuron, muscle function

## Abstract

Background and objective

The H-reflex amplitude of certain muscles, such as the wrist and ankle, decreases immediately after performing a force control task that actively controls muscle output. However, this was unknown for the fingers. Therefore, the effect of force control exercises on the excitability of anterior horn cells of the spinal cord in the thumb was investigated using F waves.

Materials and methods

The participants were 15 healthy subjects. The exercise was to control the palmar abduction of the finger on the non-dominant hand side to 20% MVC (maximum voluntary contraction: MVC). In the force control task, visual information was used to actively control muscle output. In the non-force control task, participants were instructed to hold the thumb in a palmar abduction position against a constant external load, using isometric muscle contraction to maintain the posture without visual feedback or force modulation. Each task was practiced 10 times for 90 seconds, and the F wave was derived from the short abductor pollicis brevis muscle pre- and post-assignment at rest.

The F/M amplitude ratio and frequency of appearance were used as analysis items; the F/M amplitude ratio was defined as the average of the vertex-to-vertex amplitudes of the 30 F waves obtained in one stimulus divided by the maximum amplitude value of the M wave, expressed as a percentage. Persistence was defined as the number of F waves that appeared for the number of stimuli in one trial and expressed as a percentage.

Motor performance was assessed by performing controlled movements of 20% MVC of the thumb palmar abduction pre- and post-task with visual deprivation.

Results

In the force control task, the F/M amplitude ratio decreased post-task compared to pre-task. In the non-force control task, no differences were found between pre- and post-task. There were no differences in motor performance before and after practice for both tasks.

Conclusion

In the force control task, there was a decrease in the amplitude F/M ratio post-task. Furthermore, this decrease in the amplitude F/M ratio was thought to be due to active force control.

## Introduction

In central nervous system disorders, it is essential to facilitate motor learning in order to reacquire lost movements and behaviors. Motor learning is the process of relatively permanent changes in motor performance through practice and experience, the acquisition of which requires prolonged practice [1]. On the other hand, there exists a phenomenon in which performance is temporarily enhanced by brief periods of practice, which is called “motor adaptation” and is distinguished from motor learning [2,3].

To facilitate motor learning, short-term practice must first induce immediate changes in the nervous system, some of which are thought to be related to changes in neural excitability at the spinal level, the final output pathway of movement.

The H reflex and F wave are widely used as a means of assessing spinal excitability. The H reflex is a compound electromyographic activity recorded from the homonymous muscle by stimulating Ia fibers, the afferent pathway of the stretch reflex, with electrical stimulation, thereby exciting alpha motor neurons. The F wave is a compound electromyographic activity produced when impulses conducted retrogradely through the axons of α-motor neurons are re-ignited in the anterior horn cells of the spinal cord and then conducted to the muscle in a progressive manner, and has the feature that the re-excitability of the anterior horn cells themselves can be directly assessed without involving the reflex arch [4].

Previous studies have reported improved motor performance and reduced amplitude of the H-reflex after performing a force control task that actively regulates muscle output [5,6]. Most of these studies have focused on proximal joints such as the wrist and ankle, suggesting that short-term practice may produce neural adaptations at the spinal level.

However, because the H reflex is mediated by the stretch reflex, it is susceptible to peripheral sensory input and inhibitory interneurons, limiting the ability to capture pure excitatory changes in spinal anterior horn cells. Furthermore, the H reflex is technically difficult to record from the distal muscles of the upper extremity, especially the fingers, and few studies have been conducted on the hand. On the other hand, F waves can be recorded from all muscles, making them suitable for assessing spinal excitability in distal muscles such as the fingers.

Stroke patients are prone to spasticity in the fingers, and recovery of the force-regulation function of the fingers is directly related to reacquisition of activities of daily living [7]. Therefore, clarifying the relationship between force control and spinal cord neural activity in the distal part of the upper extremity, such as the thumb, is of great clinical significance. Clarifying the relationship between motor performance and changes in spinal cord anterior horn cell excitability will also help to elucidate the causal relationship between motor performance and changes in neural excitability, or whether active control of force itself influences neural activity.

The purpose of this study was to evaluate the immediate effects of an active force control task in the thumb on spinal anterior horn cell excitability at rest immediately post-task in healthy adults using F waves. In particular, we tested the hypothesis that anterior horn cell excitability is reduced as measured by the F/M ratio and persistence. In addition, the relationship between exercise performance and measures of neural excitability was explored.

## Materials and methods

Participants

Participants were recruited by posting recruitment information on a bulletin board in Sakakibarahakuho Hospital. The details of the research and the time required were clearly indicated, and those who wished to participate were asked to contact the person in charge of the research directly. No honorarium was offered for participation. 

For the number of recruits, we conducted a power analysis using G*Power (version 3.1.9.6) prior to the study. We decided to use the corresponding t-test if the data were normally distributed and the Wilcoxon signed-rank test if the data were not normally distributed. The types of power analysis used were “a priori: compute required sample size given α, power, and effect size,” “difference between two dependent means (matched pair),” and Wilcoxon signed-rank test (matched pair)". The effect size dz = 0.8, α = 0.05, and power (1-β) = 0.80. As a result, the minimum sample size required was calculated to be 15 participants. Fifteen healthy participants (eleven men and four women, mean age 24.8 ± 3.21 years) who met the sample size requirement participated in the study.

Exclusion criteria for participants were a past or present neurological or orthopedic disease. The side examined was the non-dominant hand. A laterality coefficient of 64% or higher on the Edinburgh Handedness Inventory was used to classify a hand as dominant [8,9]. For all participants, written informed consent was provided prior to participation in the study. The ethics committee of Kansai University of Health Sciences approved the study (23-14).

Experimental setup

The experiment was conducted in a laboratory controlled at 25°C at all times. The exercise was to control maternal thumb palmar abduction on the non-dominant hand side to 20% maximal voluntary contraction (MVC). The posture was resting back supine with the non-dominant hand side in forearm pronation. The participant's MVC was measured on a separate day beforehand to ensure that the task results were not affected. MVC was measured by exerting maximum force against a compression-type load cell (TR20-100N; SOGOHKEISO, Inc., Kanagawa, Japan) with palmar abduction of the thumb of the non-dominant hand for 10 seconds [10].

**Figure 1:**
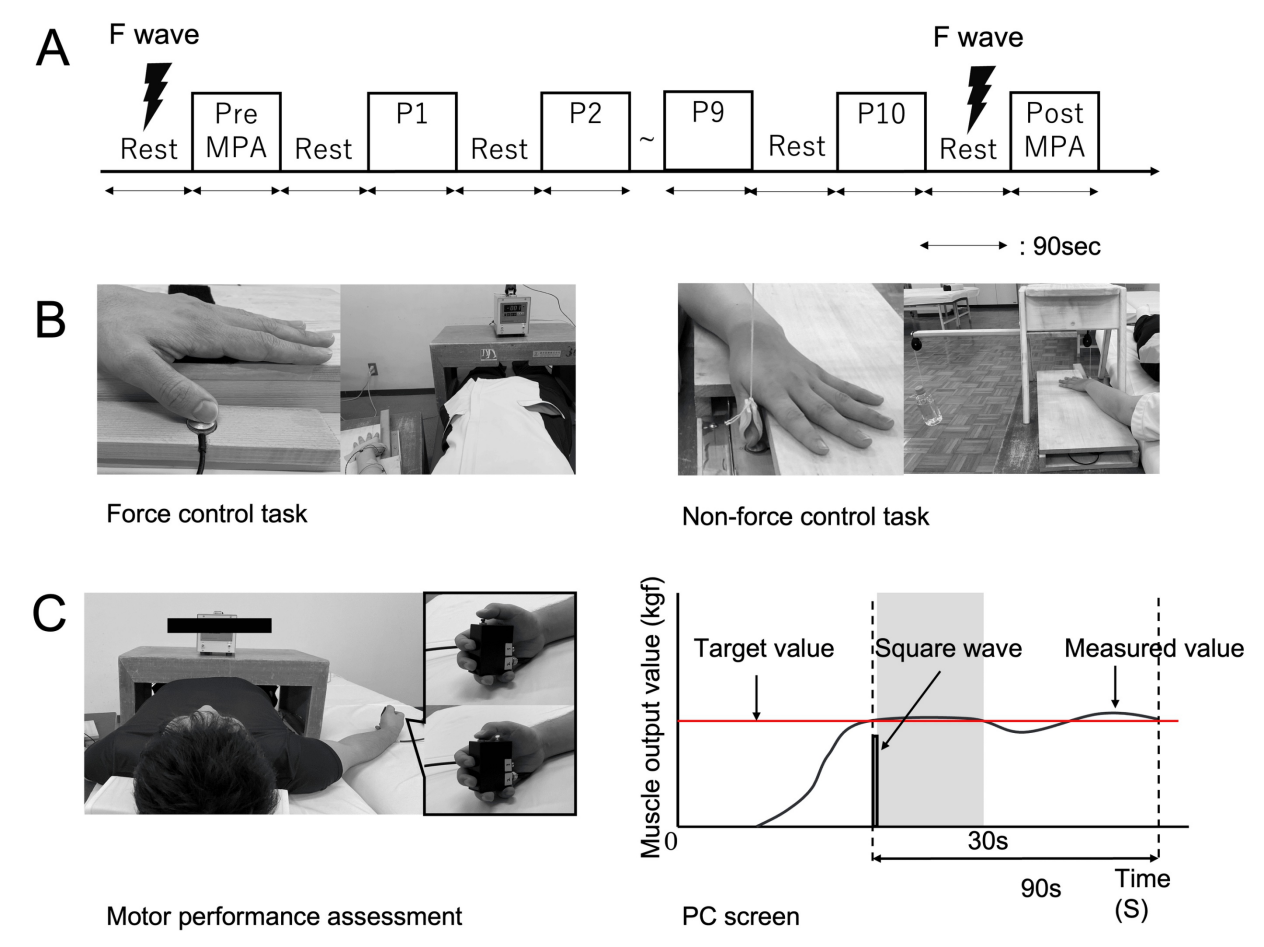
Experimental procedures MPA: motor performance assessment; P1~10: practice 1~10; MVC: maximum voluntary contraction (A) The practice consisted of 10 sessions of 90 seconds each, and F waves were obtained from the short abductor pollicis brevis muscle pre- and post-assignment at rest. Motor skills were assessed pre- and post-assignment. (B) In the force control task, the participant was asked to actively control the force to a value of 20% MVC based on visual information. In the non-force control task, the participant was given a load of 20% MVC with a dead weight. (C) A 20% MVC was maintained with the visual information hidden, and a switch was pressed once when it was determined that the 20% MVC had been reached. When the switch was pressed, a square wave appeared on the PC screen, and the exercise performance was defined as 30 seconds after its appearance.

First, the resting F wave was derived. Then, the participant was taught and pre-practiced the task to be used to evaluate motor performance. Next, the evaluation of motor performance was conducted. The number of practice sessions was 10 sessions of 90 seconds each. Finally, after practice, the resting F wave was derived, and motor performance was assessed again (Figure 1A). Two practice assignments were set (Figure 1B). The first task was a force control task in which participants actively controlled muscle output equivalent to 20% MVC while performing palmar abduction with the thumb of the non-dominant hand. The exerted muscle output was displayed numerically on a Digital Indicator (TA-802; SOGOHKEISO, Inc., Kanagawa, Japan) in real time. The second task was a non-force control task in which the thumb was pulled in the direction of palmar abduction with a load of 20% MVC, exerting muscle contraction against an externally applied load and holding the limb position of the thumb in the palmar abduction position. The difference between the tasks lies in the active or non-active control of forces. Each task was performed in a crossover design with a corresponding target population to prevent bias in individual motor skills. Furthermore, the order in which each task was performed was randomized, with a one-week washout period.

F wave measurements

An electromyography machine (Viking EDX; Nuts Medical, Inc., Pleasanton, California) was used to record F waves from the abductor pollicis brevis muscle of the non-dominant hand at rest. As recording conditions, the search electrode was placed on the muscle belly of the abductor pollicis brevis muscle of the nondominant hand, the reference electrode on the proximal phalanx of the thumb, and the ground electrode on the forearm. For the stimulation electrode, the cathode was placed on the median nerve of the wrist joint of the non-dominant hand, and the anode was placed 2 cm proximal to the cathode. Stimulation conditions were adjusted so that the stimulation intensity was 20% higher than the value at which the maximum amplitude of the M wave was obtained. The stimulation duration was 0.2 ms, and the stimulation frequency was 0.5 Hz. The bandwidth filter range was 15 Hz to 10 kHz. The sampling rate was 48 kHz. 

Various criteria have been reported in the literature for the minimum amplitude value of the F wave, such as 25 μV or greater [11] or 35 μV or greater [12]. In this study, we recognize that more detailed criteria need to be considered, but the specifications of the electromyography measurement device used (Viking EDX; Nuts Medical, Inc., Pleasanton, California) are set to exclude amplitudes of less than 30 μV. Therefore, a minimum amplitude value of 30 μV for the F wave was adopted in this study [13]. 

The F/M amplitude ratio was defined as the average of the vertex-to-vertex amplitudes of the 30 F waves obtained in one trial of stimulation divided by the maximum amplitude value of the M wave and expressed as a percentage. Persistence was defined as the number of F waves that appeared relative to the number of stimuli in a trial and was expressed as a percentage. 

To minimize bias in the analysis of F wave data, the primary author first conducted the initial waveform analysis. These data were then reviewed and corrected, if necessary, by a co-author blinded to task conditions (pre/post, force/non-force control), ensuring objective and unbiased interpretation of the electrophysiological data.

Motor performance task

Motor performance was evaluated by having participants maintain 20% MVC by performing palmar abduction with the thumb of the non-dominant hand for 90 seconds without visual feedback. Participants were instructed to press a switch held in the dominant hand once when they internally judged that they had reached and maintained 20% MVC based on their proprioceptive sense (Figure 1C).

Prior to the motor performance evaluation task, participants underwent practice sessions during which visual feedback was provided to help them learn and familiarize themselves with the sensation of exerting 20% MVC. In the actual evaluation task, participants adjusted their force based solely on this internally acquired proprioceptive sense, without visual information.

 Electromyogram recording software (Vital Recorder 2; KISSEI COMTEC, Inc., Nagano, Japan) recorded muscle output values exerted from the thumb of the non-dominant hand to a compression-type load cell. The sampling frequency was 1000 Hz. The versatile bioanalysis system (BIMUTAS-Video; KISSEI COMTEC, Inc., Nagano, Japan) was used to analyze muscle output values. The values input from the pinch sensor were displayed on the PC screen, and a switch was set to cause a square wave to appear on the PC.

The absolute error and the coefficient of variation of the absolute error between the muscle output value being exerted for 30 seconds from the time of appearance of this square wave and the defined 20% MVC for each participant were calculated as an index of motor skill.

Data analysis

Statistical analysis was performed using R version 4.3.3 (R Foundation for Statistical Computing, Vienna, Austria). M-wave amplitudes were analyzed pre- and post-each task. These were to ensure that the position of the stimulating electrode on the skin over the median nerve would not change. Normality was assessed with the Shapiro-Wilk test, but since it was not normally distributed, the Wilcoxon signed-rank test was used. The F/M amplitude ratio and persistence were analyzed pre- and post-each task. An assessment of normality was performed with the Shapiro-Wilk test, but since it was not normally distributed, the Wilcoxon signed-rank test was used. Absolute error and coefficient of variation of absolute error were analyzed pre- and post-each assignment. The Shapiro-Wilk test was used to assess normality, but since it was not normally distributed, the Wilcoxon signed-rank test was used. Correlations were analyzed to determine the association between changes in spinal anterior horn cell excitability and changes in motor skills. First, the Shapiro-Wilk test was used to assess the normality of all values. Since all data were not normally distributed, Spearman's rank correlation coefficient was used. The significance level was set at 5%.

## Results

Changes in M wave amplitudes

The amplitude of the M wave in the force control task was 17.6 mV (IQR: 13.6-20.2) pre-task and 17.7 mV (IQR: 13.8-20.2) post-task, with no difference between pre-and post-task (*p* = 0.42, d = 0.07). The amplitude of the M wave in the non-force control task was 16.9 mV (IQR: 13.3-18.6) pre- and 17.5 mV (IQR: 14.0-18.3) post-task, with no difference between pre-and post-task (*p* = 0.55, d = 0.13).

Changes in excitability of spinal cord anterior horn cells

**Figure 2:**
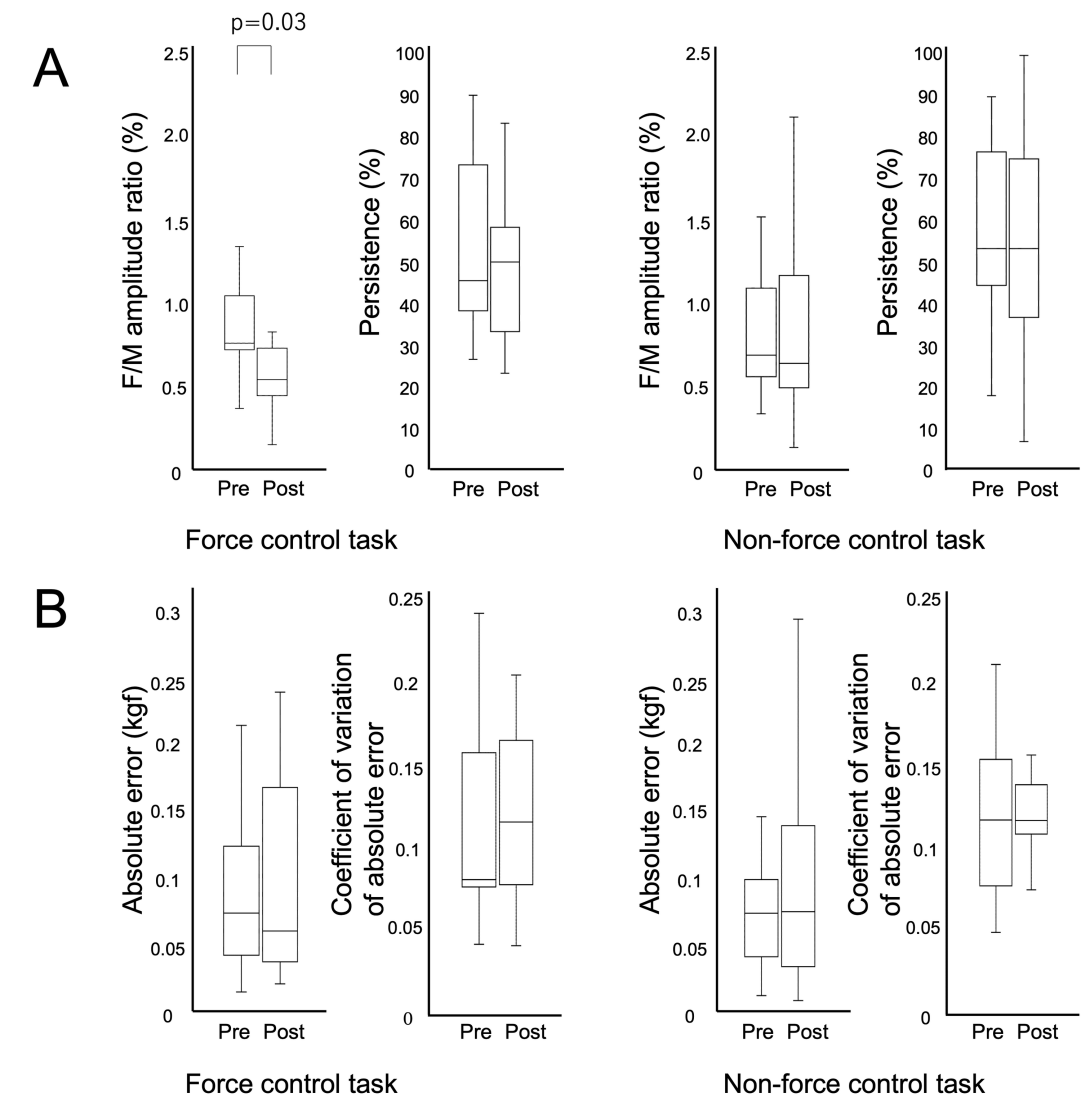
Changes in spinal cord anterior horn cell excitability and motor performance Pre: pre-task, post: post-task (A) In the force control task, the F/M amplitude ratio decreased post-task compared to pre-task. (B) Motor performance did not differ before and after practice in both tasks. Analysis: Wilcoxon signed-rank test

The F/M amplitude ratio of the force control task was 0.76 % (IQR: 0.73-1.04) pre-task and 0.55 % (IQR: 0.45-0.73) post-task, decreasing post-task compared to pre-task (*p*=0.03, d=0.85). The persistence of the force control task was 45.6 % (IQR: 38.3-73.3) pre-task and 50.0 % (IQR: 33.3-58.3) post-task, with no difference between pre-and post-task (*p*=0.17, d=0.31) (Figure 2A).

**Figure 3:**
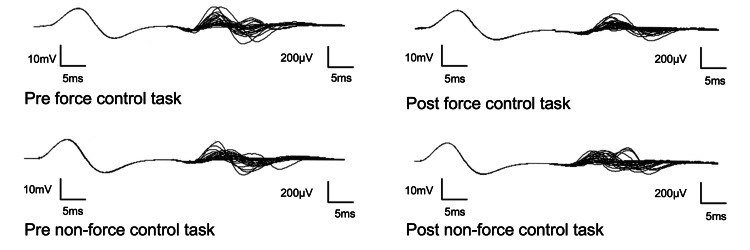
A typical F wave

The F/M amplitude ratio for the non-force control task was 0.69 % (IQR: 0.56-1.09) pre-task and 0.64 % (IQR: 0.49-1.17) post-task, with no difference between pre- and post-task (*p *= 0.12, d = 0.07). The persistence of the non-force control task was 53.3 % (IQR: 44.4-76.7) pre- and 53.3 % (IQR: 36.7-75.0) post-task, with no difference between pre-and post-task (*p*=0.32, d=0.16) (Figure 2A). A typical F waveform is shown in Figure 3.

Changes in motor performance

The absolute error for the force control task was 0.07 kgf (IQR: 0.04-0.12) pre- and 0.06 kgf (IQR: 0.04-0.17) post-task and did not differ pre- and post-task (*p *= 0.93, d = 0.23). The coefficient of variation of absolute error for the force control task was 0.08 (IQR: 0.08-0.16) pre- and 0.12 (IQR: 0.08-0.16) post-task and did not differ pre- and post-task (*p* = 0.89, d = 0.08) (Figure 2B).

The absolute error in the non-force control task was 0.07 kgf (IQR: 0.04-0.10) pre- and 0.07 kgf (IQR: 0.06-0.14) post-task and did not differ pre- and post-task (*p* = 0.45, d = 0.38). The coefficient of variation of absolute error in the non-force control task was 0.10 (IQR: 0.06-0.14) pre- and 0.10 (IQR: 0.09-0.12) post-task, with no difference between pre-and post-task (*p* = 0.64, d = 0.23) (Figure 2B).

Relationship between the F/M amplitude ratio and changes in motor performance

**Figure 4:**
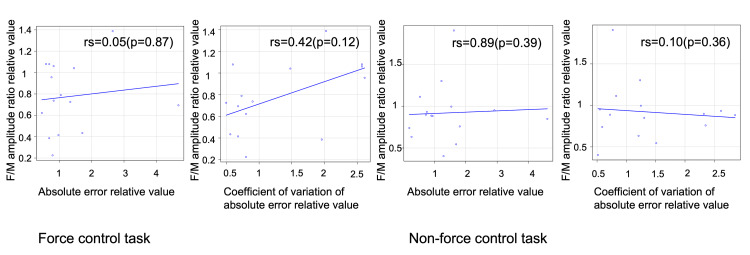
Correlation between the F/M amplitude ratio and exercise performance F/M amplitude ratio relative value: normalized relative value of the post-task resting F wave value to the pre-task resting F wave value; absolute error relative value: normalized relative value of the post-task absolute error value to the pre-task absolute error value; coefficient of variation of absolute error relative value: normalized relative value of the pre-task coefficient of variation to the post-task coefficient of variation. Analysis: Spearman's correlation coefficient.

No correlation was found between the F/M amplitude ratio and absolute error in the force control task (r_s_=0.05, *p*=0.87). No correlation was also found between the F/M amplitude ratio and coefficient of variation in the force control task (r_s_=0.42, *p*=0.12) (Figure 4).

No correlation was found between the F/M amplitude ratio and absolute error in the non-force control task (r_s_=089, *p*=0.39). There was also no correlation between the F/M amplitude ratio and coefficient of variation in the non-force control task (r_s_=0.10, *p*=0.36) (Figure 4).

## Discussion

Changes in spinal cord anterior horn cells immediately post-task due to the force control task 

In the force control task, the amplitude of the F/M ratio decreased post-task compared to pre-task, while persistence remained unchanged. The M-wave amplitude also remained stable across measurements, suggesting that the reduction in the F/M ratio was not due to changes in peripheral excitability at the muscle level but may instead reflect altered excitability of spinal anterior horn cells.

The F/M ratio is believed to be affected by the size of the refiring motoneurons and the degree of synchronization. Therefore, we speculate that the reduction in the F/M ratio observed in the present study is due to selective suppression of the excitability of larger motor units by the performance of an active force control task or a change in the timing of motor neuron reactivation. On the other hand, the fact that the F wave persistence rate was maintained pre- and post-task indicates that the total number of motor units that responded did not change, speculating that the decrease in the F/M ratio was due to a change in the composition of the motor units that fired, i.e., increased involvement of relatively small motor units.

Prior reports of H reflex reduction following similar tasks [5,6,14] have been interpreted as reflecting enhanced inhibitory interneuron activity, which may persist after task completion. Although our study did not directly assess inhibitory interneuron activity, the decrease in the F/M ratio may be related to similar inhibitory mechanisms at the spinal level.

The absence of changes in either the F/M amplitude ratio or persistence in the non-force control task condition supports the notion that active engagement in force modulation plays a role in modulating spinal motoneuron excitability.

It should be noted that this study focused on the short-term and immediate effects of the thumb force control task on the excitability of anterior horn cells in the spinal cord, and it is not clear whether the changes obtained are long-lasting or lead to long-term neuroplasticity. This is one of the limitations of this study, and future studies should examine whether there are long-term effects through the implementation and evaluation of ongoing tasks over time. Furthermore, because this study did not use direct measures of cortical excitability such as motor evoked potentials (MEPs), it is not possible to clearly distinguish whether the changes observed in the present study were due to changes at the spinal level or to changes of central origin, such as the cerebral cortex. This is another limitation of the present study, and future studies should examine in more detail the central and peripheral contributions to the neural mechanisms that occur after voluntary force control of the thumb by combining techniques such as transcranial magnetic stimulation.

Changes in motor performance by force control

Motor performance did not improve between the two tasks in this study. Active force control tasks based on visual information have been reported to improve motor performance [15-17]. However, these studies used tasks with variable target intensity. It has been reported that whether or not exercise performance is improved depends on the complexity of the task [18]. Therefore, we considered that this study may have been an easy task and did not show any change in motor performance.

Correlation between force-controlled motor performance and the F/M amplitude ratio

In the present study, no significant association was found between spinal anterior horn cell excitability and motor performance. This result aligns with previous reports suggesting that the temporal relationship between motor performance improvements and changes in central nervous system excitability is not always consistent. For example, in a force control task involving index finger abduction, performance improvements were observed prior to detectable changes in corticospinal excitability as measured by MEPs [16]. Similarly, a reduction in the H reflex of the flexor carpi radialis muscle following wrist force control training was reported to occur during the acquisition phase of motor performance [6], indicating that neurophysiological adaptation and performance improvement do not necessarily occur in parallel.

These findings support the idea that motor performance and spinal excitability may be partially dissociable processes, influenced by different aspects of motor control and learning. In the present study, the modulation of anterior horn cell excitability following the thumb force control task may reflect task-specific neural adaptation, especially due to the precise force regulation required by distal hand muscles, rather than being directly linked to motor performance improvements or general muscle activation.

Additionally, variability in task difficulty, practice time and frequency and individual differences in motor control strategies may have influenced the lack of correlation observed. Further research is needed to elucidate how these factors interact in shaping both motor performance and spinal excitability.

## Conclusions

This study investigated the immediate neurophysiological changes induced by a voluntary force control task of the abductor pollicis brevis muscle in healthy adults. The results demonstrated that the reduction in the F/M amplitude ratio was not simply attributable to task execution itself, but rather to the performance of the force control component of the task.

This finding suggests that specific modulation occurred in the excitability of spinal anterior horn cells and that such neurophysiological changes may proceed through mechanisms distinct from those underlying improvements in motor performance. Taken together, the execution of tasks involving fine force control may contribute to the modulation of excitability in spinal motoneurons. While these findings provide preliminary insights, further research is necessary to determine whether such neuromodulatory effects can be translated into clinically meaningful outcomes, such as the modulation of abnormal muscle tone.
